# Chronic Effects of Pediatric Ear Infections on Postural Stability

**DOI:** 10.1155/2021/6688991

**Published:** 2021-02-13

**Authors:** Ohud A. Sabir, Eric G. Johnson, Ammar E. Hafiz, Rhonda N. Nelson, Mitali Hudlikar, Isha Sheth, Noha S. Daher

**Affiliations:** ^1^Department of Physical Therapy, Faculty of Medical Rehabilitation Sciences, King Abdulaziz University, Jeddah, Saudi Arabia; ^2^Department of Physical Therapy, Loma Linda University, California, USA; ^3^Department of Allied Health Studies, Loma Linda University, California, USA

## Abstract

**Background:**

Ear infections in children often cause abnormal postural stability. However, the long-term effects of recurrent ear infections on postural stability have not been investigated.

**Purpose:**

The purpose of this study was to examine the long-term effects of multiple ear infections on pediatric postural stability.

**Methods:**

Forty children aged 10-12 years were divided into two groups (18 participants with a history of tympanostomy tubes and/or 3 or more ear infections prior to age five and 22 participants without a history of tympanostomy tubes and/or 0-2 ear infections prior to age five). Computerized Stability Evaluation Test (SET) and noncomputerized postural stability were measured for all participants.

**Results:**

A significant difference was found in median postural stability scores in the SET during a tandem stance on an unstable surface between the two groups (median (minimum, maximum) of 9.1 (1.4, 11.4) versus 5.8 (1.7, 12.8), *p* = 0.04). In addition, there was a significant difference in median Pediatric Balance Scale scores between participants with versus without ear infection (54 (47, 56) versus 56 (55, 56), *p* = 0.001).

**Conclusions:**

Results suggest that children ages 10-12 with a history of tympanostomy tubes and/or 3 or more ear infections prior to age five have decreased postural stability.

## 1. Introduction

Otitis media with effusion (OME), or “glue ear,” has been defined as a serous to thick fluid in the middle ear without acute infection symptoms that can cause temporary hearing loss [1–3]. Otitis media with effusion is considered a common cause of vestibular disturbance and movement disorganization in pediatrics [4]. However, there are a small number of studies on the relationship between vestibular hypofunction and OME in pediatrics [4]. Chronic OME has been identified as a principal cause of balance instabilities and vertigo in pediatrics [5, 6].

Rosenfeld et al. [2] suggested that the persistence of fluid in the middle ear, which comes from OME, might lead to reduced tympanic membrane mobility and negatively affect sound conduction. Approximately 25% of chronic OME episodes lasting more than three months negatively affect hearing, vestibular function, behavior, school performance, and quality of life [2]. The effect of OME on postural stability in children has been assessed by many investigators; however, none of these studies analyzed postural stability changes depending on the functional condition of the middle ear [6]. Middle ear dysfunction includes partial or complete blockage of the Eustachian tube that contributes to chronic ear infections and failure to effectively regulate ear pressure [7].

Recent research shows that during recurrent OME episodes, children have abnormal balance and vestibular hypofunction [8]. Because OME is the most common disease in pediatrics, it is important to examine their postural stability, vestibular system, and motor function during both the presence and absence of episodes to know if the child is at risk for these problems [8]. The primary reason parents of children with OME consult an otolaryngologist is hearing loss, which is the most common complication of OME [9]. Williamson [3] determined that 91% of children between the ages of two months and two years will experience one episode of OME, and 52% of children will have bilateral middle ear effusion [3]. Risk factors that have been described in case-control studies include attendance at day care centers, children age six years and younger, exposure to second-hand smoking, repeated upper respiratory tract infections, and a significant number of siblings [3].

Robb and Williamson [1] reported that there is a bimodal peak of incidence in children aged between two and five years old, with OME episodes resolving spontaneously within three months in approximately half of the cases. The vestibular, visual, and proprioceptive systems are the main components providing postural stability [5]. When one of the three postural stability systems becomes dysfunctional, vestibular compensatory efforts are increased [5]. The structural complications in children with middle ear effusion are present in the tympanic membrane and ossicular chain of the ear [10]. Reddy [11] reported that the lack of Eustachian tube ventilation plays an important role in developing OME. Eustachian tube dysfunction is the lack of ability of the Eustachian tube to sufficiently perform at the minimum one of its functions, which are ear protection, middle ear ventilation, and mucociliary clearance of the middle ear. The impairment of middle ear pressure regulation and ventilation due to Eustachian tube obstruction leads to increase negative pressure in the middle ear. The angle and length of the Eustachian tube are more horizontal and shorter in infants than in adults. The angle becomes more vertical and longer with age as children grow, and these developmental changes are greatest between the ages of three to four years [12]. Takasaki et al. [12] reported that there was no statistical difference between the angle and length of the Eustachian tube in infants with and without OME.

Rosenfeld et al. [2] reported that 90% of children have OME before they are of school age and have, on average, one episode annually. This demonstrates how common this disease is in pediatrics and is why it is now called an “occupational hazard of early childhood” [2].

Postural stability and balance are important components for purposeful and functional movement [13]. The mechanism of how OME affects postural stability has been explained as subsequent changes in the ionic channels of the kinocilia and stereocilia by the transfer of ions through the semipermeable membrane gate which affect balance [5]. This transfer changes the normal chemical composition of the endolymph and perilymph within the inner ear, which can impact balance [5]. Children learn by interacting with and exploring their environment [13].

Casselbrant et al. [14] suggested that children with OME who had vestibular hypofunction may solely rely upon the visual and proprioceptive systems to support postural stability. Postural sway in children with OME is greater than in children without OME when they respond to a visually moving object [14]. To identify the importance of the vestibular system in managing OME in pediatrics, further studies are needed [14].

Postural stability and the vestibular system in children with recurrent OME may or may not completely improve after the disease resolves [9]. In order to avoid complications in the child's developing sensory integration, early intervention should be considered [9].

Current OME clinical practice guidelines strongly recommend tympanostomy tube insertion to manage children with OME lasting three months or longer [2]. This procedure involves surgical tube placement through a myringotomy incision to ventilate middle ear pressure [15]. It is the most common ambulatory surgery performed in the United States as 667,000 children younger than 15 years old undergo the surgery annually [15].

Our aims in this study were to examine the effect of multiple ear infections on pediatric postural stability and visual overreliance. We hypothesized that postural stability would be worse in children with a history of multiple ear infections compared to children without a history of multiple ear infections and children with a history of multiple ear infections would be more visually dependent for maintaining postural stability than those without a history of multiple ear infections.

## 2. Methods

Forty children aged 10-12 years with and without a history of multiple ear infections and/or tympanostomy tubes prior to age five were recruited for this study from the local community. Based on parent self-report to a single question, participants were divided into two groups (18 participants with a history of tympanostomy tubes and/or 3 or more ear infections prior to age five and 22 participants without a history of tympanostomy tubes and 0-2 ear infections prior to age five). Participants were excluded if they had disorders of balance and gait, seizure disorders, noncorrected visual deficits, and/or medications affecting balance. Parents of all participants signed Loma Linda University's Institutional Review Board approved informed consent prior to study participation, and then, the children read and signed the informed assent.

The Physical Activity Questionnaire for Children (PAQ-C) was used to assess the general activity level for the children. The PAQ-C was developed for elementary school-aged children ages 8-14 years and is a nine-item questionnaire pertaining to recollection of the child's last seven days of physical activity. The total score was calculated by taking the mean of the 9 items. A score of one indicated a low physical activity, while a score of five indicated a high physical activity level for the child [16]. Voss et al. [17] reported that the PAQ-C is a valid and reliable tool used to assess physical activity level in elementary school-aged healthy children.

Postural stability was measured using three different assessment tools: The Bertec Balance Advantage Dynamic Computerized Dynamic Posturography with Immersion Virtual Reality (CDP-IVR), Pediatric Balance Scale (PBS), and the NeuroCom® VSR™ SPORT Computerized Dynamic Posturography Stability Evaluation Test (SET). The CDP-IVR measured static postural stability by calculating participants' center of gravity displacements under three conditions [18]. Condition 1 measured the postural stability baseline using a stable force plate with eyes open. Condition 2 measured postural stability on a stable force plate with eyes open and focusing on a virtual reality infinite tunnel. Condition 3 measured postural stability on an unstable force plate with eyes closed. Each condition included three 20-second trials, and the average of the three trials for each condition was calculated. The CDP-IVR calculates postural stability and generates an equilibrium score in the following manner: signals from the participants' effort to maintain balance were sampled and analyzed at 1000 Hertz and the sway path was computed. The testing protocol calculates the sway path with equilibrium scores quantified by how well the participants' sway remains within the expected angular limits of stability during each testing condition. The following formula was used to calculate the equilibrium score: equilibrium score (ES) = ([12.5 degrees − (the taMAX–the taMIN)]/12.5 degrees)∗100. The ES uses 12.5° as the normal limit of the anterior-posterior sway angle range, taMAX is theta maximum, and taMIN is theta minimum. Sway angle was calculated as follows: sway angle = arcsin (COG*y*/(.55∗*h*)), where *y* is the anterior-posterior sway axis and *h* is the participant's height in cm or inches. The inverse Sin of the center of gravity was divided by 55% of the subject's height. Participants exhibiting little sway achieve equilibrium scores near 100, while participants exhibiting more sway achieve equilibrium scores further away from 100 [19].

The PBS was used to assess balance in participants. The PBS is a noncomputerized 14-item criterion-referenced tool that measures the static and dynamic functional balance of daily activities in children [19, 20]. The PBS tasks include items such as sit to stand, transfers, and turning 360 degrees [19, 20]. Yi et al. [21] reported that the PBS is a simple and valid tool that could be used in pediatric rehabilitation to examine functional balance and predict motor capability and capacity. In addition, Chen et al. [22] reported that the PBS is a valid tool to examine functional balance in children with cerebral palsy. Franjoine et al. [23] reported that the PBS is a reliable tool to assess balance in school-aged children with mild to moderate motor impairments.

Postural stability was also measured using SET. The SET has good to excellent reliability for postural stability measurement (ICC = 0.93; 95% CI: 0.88, 0.95) with significant practice effects (*p* < 0.05) [24]. Static postural stability was tested on a stable surface in three conditions. SET Condition 1 measured postural stability with a narrow double limb stance on a firm surface. SET Condition 2 measured postural stability with a single limb stance on a firm surface. SET Condition 3 measured postural stability with a tandem (dominant foot behind) stance on a firm surface. SET Condition 4 measured postural stability with a narrow double limb stance on an unstable surface. SET Condition 5 measured postural stability with a single limb stance on an unstable surface. SET Condition 6 measured postural stability with a tandem (dominant foot behind) stance on an unstable surface. In all conditions, participants closed their eyes and placed their hands on their iliac crests. The SET protocol quantifies the center of gravity sway or postural stability as a weighted average of all six conditions.

## 3. Procedures

Participants completed the PAQ-C. Before postural stability testing, participants removed shoes and socks followed by height and weight measurements. Participants donned a safety harness before postural stability was measured. Postural stability was measured using three different assessment tools. Postural stability was first measured using the CDP-IVR in the three conditions described above. Next, the participant's postural stability was measured using the PBS. Each item ranged from 0 (unable to perform) to 4 (able to perform the task without difficulty independently as instructed). Participants were given verbal and visual instructions for each item. One trial was given to each participant. A second trial was given if the participant was unable to understand verbal or visual instructions. Finally, postural stability was measured using the SET, which measured static postural stability on a stable surface in six conditions including a double limb stance, single limb stance, and tandem (dominant foot behind) stance. In each condition, participants closed their eyes and placed hands on their iliac crests. The same three conditions were repeated on a foam surface to test postural stability on an unstable surface.

Data analysis was performed using the Statistical Package for the Social Sciences (SPSS) version 24.0 (IBM Corp, Armonk, NY). A sample size of 42 subjects was estimated using an effect size (*d* = 0.90), a power of 0.80, and a level of significance set at 0.05. The general characteristics of the participants were summarized using means and standard deviations for quantitative variables and counts and percentages for categorical variables. The frequency distribution of gender was compared between the two study groups using the chi-square test. The normality of the continuous variables was examined using the Shapiro-Wilk test and box plots. We compared mean age, BMI, and postural stability during conditions one, two, and three, double leg stance on stable and foam surfaces, single leg stance on stable and foam surfaces, and tandem stance on a stable surface using the independent *t*-test. The distribution of postural stability during a tandem stance on a foam surface and PBS scores were not approximately normal, and thus, the Mann-Whitney test was used to examine differences in median stability scores between the two groups. The level of significance was set at *p* ≤ 0.05.

## 4. Results

Forty children (20 males and 20 females) with mean age 10.7 ± 0.8 years participated in this study. The study sample comprised of 18 children with a history of tympanostomy tubes and/or 3 or more ear infections and 22 children without a history of tympanostomy tubes and/or 0-2 ear infections. The baseline characteristics were similar between the two groups (*p* > 0.05). There was no significant difference in mean postural stability in CDP-IVR at baseline, during eyes open, eyes closed, and immersion virtual reality conditions. Also, no significant differences in mean postural stability in the SET during a double leg stance on firm and unstable surfaces, single leg stance on firm and unstable surfaces, and tandem stance on a firm surface were observed (*p* > 0.05, Table 1).

**Figure 1:**
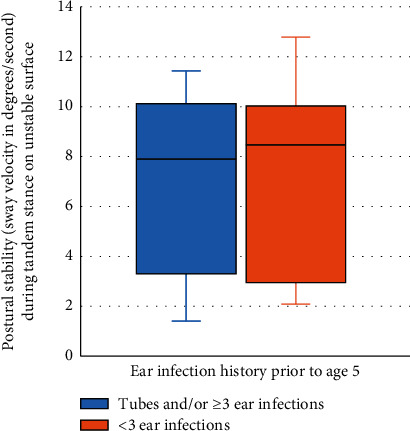
SET postural stability scores during a tandem stance on an unstable surface by the study group.

**Figure 2:**
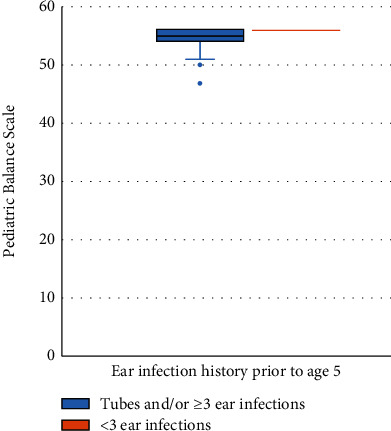
Pediatric Balance Scale score by the study group.

A significant difference was found in median postural stability scores in the SET during tandem stance on an unstable surface between the two groups (median (minimum, maximum) of 9.1 (1.4, 11.4) versus 5.8 (1.7, 12.8), *p* = 0.04; Figure 1). In addition, there was a significant difference in median PBS scores between participants with versus without ear infection (54 (47, 56) versus 56 (55, 56), *p* = 0.001; Figure 2).

## 5. Discussion

The present study investigated the chronic effects of pediatric ear infections, prior to age five, on postural stability in children aged 10-12 years with a history of tympanostomy tubes and/or 3 or more ear infections versus those without a history of tympanostomy tubes and/or 0-2 ear infections by using the CDP-IVR, SET, and PBS. Our results determined that tandem stance in both the SET and the PBS caused the most postural instability in the children with a history of tympanostomy tubes and/or 3 or more ear infections. Moreover, tandem stance with eyes closed on an unstable surface proved most challenging for children with a history of tympanostomy tubes and/or 3 or more ear infections.

Casselbrant et al. [8] reported that during recurrent OME episodes, children have abnormal balance and vestibular hypofunction. In addition, postural stability and the vestibular system in children with recurrent OME may or may not completely improve after the OME episode resolves [9]. The present study results support those findings and suggest that postural stability did not improve completely after the resolution of OME.

Casselbrant et al. [14] suggested that children with OME and vestibular hypofunction might solely rely on the visual and proprioceptive systems to support postural stability. They used a posture platform with a visual surround (EquiTest, NeuroCom, Inc.) in three conditions: no visual scene movement, scene movement at 0.10 Hz, and scene movement at 0.25 Hz to test visual balance dependency. In the present study, there was no significant difference in mean postural stability during eyes closed or during the immersion virtual reality conditions. Our results suggest that children with OME do not overrely on their visual system to compensate for their abnormal postural stability. This could be related to the fact that all participants were active as measured by the PAQ-C. However, children with multiple ear infection history that have lower activity levels might present differently [25, 26].

The CDP-IVR measures static postural stability by calculating participants' center of gravity displacements under three conditions [18]. The SET measures postural stability as sway velocity in degrees per second across several different testing conditions [27]. Participants assume a wider base of support during the CDP-IVR compared to the SET protocol, which could be the reason for the different results between the SET and the CDP-IVR. In this study, there was no significant difference in mean postural stability during the CDP-IVR eyes closed or immersion virtual reality conditions but differences were found between groups during the SET protocol.

This study had several limitations. The study sample included few participants with a history of tympanostomy tubes. Also, group assignment was based on self-report. In addition, blinding was not utilized during the PBS, which introduces the possibility of examiner bias. Future investigators should consider recruiting children with a history of tympanostomy tubes, children with multiple ear infection history that have low activity levels, and having participants' parents supply medical records regarding the history of tympanostomy tubes and ear infections. In addition, participants in both groups were physically active. Future investigators should consider recruiting participants with lower activity levels as physical activity level may positively impact postural stability in children with tubes and/or 3 or more ear infections prior to age five.

In conclusion, the results of this study suggest that children ages 10-12 with a history of tympanostomy tubes and/or 3 or more ear infections prior to age five have decreased postural stability compared to children without a history of tympanostomy tubes and/or 0-2 ear infections. The results suggest that postural stability impairments persist even after the resolution of the ear infections in early childhood. Future studies should evaluate the effects of postural stability exercises on children with a history of tympanostomy tubes and/or 3 or more ear infections prior to age five.

## Figures and Tables

**Table 1:** Mean (SD) of characteristics and outcomes by the study group (*N* = 40).

	Tubes and/or ≥3 ear infections (*n*1 = 18)	No tubes and <3 ear infections (*n*2 = 22)	*p* value (*d*)
Female, *n* (%)	11 (61.1)	9 (40.9)	0.17
Age (years)	10.5 (8.1)	10.9 (0.9)	0.17 (0.07)
BMI (kg/m^2^)	20.5 (4.9)	20.8 (4.8)	0.90 (0.06)
CDP-IVR Condition 1	89.7 (3.8)	89.4 (3.3)	0.74 (0.08)
CDP-IVR Condition 2	85.8 (5.5)	87.4 (3.5)	0.26 (0.35)
CDP-IVR Condition 3	29.9 (24.3)	31.2 (22.8)	0.87 (0.06)
SET Condition 1	0.9 (0.2)	0.8 (0.2)	0.40 (0.27)
SET Condition 4	2.1 (0.5)	2.2 (0.8)	0.65 (0.15)
SET Condition 2	4.3 (1.8)	3.9 (2.0)	0.59 (0.21)
SET Condition 5	6.3 (1.3)	5.9 (1.5)	0.46 (0.28)
SET Condition 3	3.0 (2.3)	2.8 (1.8)	0.76 (0.10)
SET Condition 6^∗∗^	9.1 (1.4, 11.4)	5.8 (1.7, 12.8)	0.04^∗^ (0.56)
PAQ-C	3.2 (0.8)	3.1 (1.0)	0.58 (0.11)
PBS^∗∗^	54 (47, 56)	56 (55, 56)	0.001^∗^ (1.33)

Abbreviation: SD: standard deviation; BMI: body mass index; PAQ-C: Physical Activity Questionnaire for Children; CDP-IVR: The Bertec Balance Advantage Dynamic Computerized Dynamic Posturography with Immersion Virtual Reality; CDP-IVR Condition 1: baseline using a stable force plate, eyes open; CDP-IVR Condition 2: stable force plate, eyes open, and focusing on a virtual reality infinite tunnel; CDP-IVR Condition 3: unstable force plate, eyes closed; SET: Stability Evaluation Test; SET Condition 1: measured postural stability with narrow double limb stance on firm surface; SET Condition 2: measured postural stability with single limb stance on firm surface; SET Condition 3: measured postural stability with tandem (dominant foot behind) stance on firm surface; SET Condition 4: measured postural stability with narrow double limb stance on unstable surface; SET Condition 5: measured postural stability with single limb stance on unstable surface; SET Condition 6: measured postural stability with tandem (dominant foot behind) stance on unstable surface. In all conditions, participants closed their eyes and placed their hands on their iliac crests; PBS: Pediatric Balance Scale. ^∗^*p* < 0.05. ^∗∗^Results are reported as median (minimum, maximum).

## Data Availability

I included all the data supporting the results of our study. If you need anything else, you can email me.
